# Organ Explant Culture of Neonatal Rat Ventricles: A New Model to Study Gene and Cell Therapy

**DOI:** 10.1371/journal.pone.0059290

**Published:** 2013-03-13

**Authors:** A. Dénise den Haan, Marieke W. Veldkamp, Diane Bakker, Geert J. J. Boink, Rob B. Janssen, Jacques M. T. de Bakker, Hanno L. Tan

**Affiliations:** 1 Heart Center, Academic Medical Center, University of Amsterdam, Amsterdam, The Netherlands; 2 Interuniversity Cardiology Institute of the Netherlands, Utrecht, The Netherlands; Centro Cardiologico Monzino, Italy

## Abstract

Testing cardiac gene and cell therapies *in vitro* requires a tissue substrate that survives for several days in culture while maintaining its physiological properties. The purpose of this study was to test whether culture of intact cardiac tissue of neonatal rat ventricles (organ explant culture) may be used as a model to study gene and cell therapy. We compared (immuno) histology and electrophysiology of organ explant cultures to both freshly isolated neonatal rat ventricular tissue and monolayers. (Immuno) histologic studies showed that organ explant cultures retained their fiber orientation, and that expression patterns of α-actinin, connexin-43, and α-smooth muscle actin did not change during culture. Intracellular voltage recordings showed that spontaneous beating was rare in organ explant cultures (20%) and freshly isolated tissue (17%), but common (82%) in monolayers. Accordingly, resting membrane potential was -83.9±4.4 mV in organ explant cultures, −80.5±3.5 mV in freshly isolated tissue, and −60.9±4.3 mV in monolayers. Conduction velocity, measured by optical mapping, was 18.2±1.0 cm/s in organ explant cultures, 18.0±1.2 cm/s in freshly isolated tissue, and 24.3±0.7 cm/s in monolayers. We found no differences in action potential duration (APD) between organ explant cultures and freshly isolated tissue, while APD of monolayers was prolonged (APD at 70% repolarization 88.8±7.8, 79.1±2.9, and 134.0±4.5 ms, respectively). Organ explant cultures and freshly isolated tissue could be paced up to frequencies within the normal range for neonatal rat (CL 150 ms), while monolayers could not. Successful lentiviral (LV) transduction was shown via *Egfp* gene transfer. Co-culture of organ explant cultures with spontaneously beating cardiomyocytes increased the occurrence of spontaneous beating activity of organ explant cultures to 86%. We conclude that organ explant cultures of neonatal rat ventricle are structurally and electrophysiologically similar to freshly isolated tissue and a suitable new model to study the effects of gene and cell therapy.

## Introduction

New therapies for various cardiovascular diseases are currently being developed with a focus on gene and cell therapy. For gene therapy, lentiviruses are attractive vectors because they are capable of transducing non-replicating cells [1] (e.g., cardiomyocytes), stably integrating the gene of interest into the host genome [2], and carrying relatively large genes [3]. The expression of lentivirally-transduced genes requires several days. This time is needed to allow the viral mRNA to be reverse transcribed into DNA, which is required for subsequent integration and expression. Evaluating the effects of cell therapy may also require several days [4]. Experimental models to test gene and cell therapy for cardiovascular diseases should therefore replicate the *in vivo* situation over a period of at least days. Although this may be achieved via animal studies, these studies have important drawbacks, including their high cost and labor intensity. Therefore, suitable *in vitro* models are important, especially as an initial strategy screening tool. While current *in vitro* models, such as cultured neonatal rat ventricular cardiomyocytes, provide the opportunity of testing cells over 1–2 weeks, important properties of the *in vivo* situation are lost in this system. For instance, neonatal rat ventricular cardiomyocytes cultured in a syncytium (monolayer) show a flat, star-shaped morphology instead of the normal rod-like shape, along with a reduction in peak transient outward current, sodium current, and calcium current [5]. Furthermore, these cells display a depolarized resting membrane potential (RMP), a reduced action potential upstroke velocity, and spontaneous beating [6], [7]. These properties are correlated to the loss of *I*_K1_ [8] and the number of myofibroblasts present in these cultures [9]. Miragoli et al. described a fast phenotypic switch from fibroblasts to myofibroblasts when in culture [10]. These myofibroblasts expressed both connexin-43 and connexin-45 at contact sites between each other and with cardiomyocytes. Since myofibroblast have a more depolarized membrane potential, coupling between myocyte and myofibroblast is believed to induce depolarization in the cardiomyocyte, thereby causing depolarization-induced automaticity [11].

Several studies describe methods to limit the changes due to cell culturing, including anisotropic growth and the use of pharmacologic agents and cell separation to reduce the number of (myo) fibroblasts [10], [12]. However, a 3-dimensional system would still be preferable over a 2-dimensional cell culture for various reasons, including differences in gene expression, regulation of cell growth and fibroblast morphology [13]. Furthermore, the 3-dimensional structure more closely resembles the *in vivo* situation and may be particularly relevant when studying electrical phenomena such as current-to-load mismatch [14].

Clearly, the development of a 3-dimensional cardiac *in vitro* model that remains viable for several days, yet maintains its electrophysiologic properties, is crucial to reliably study various gene- and cell-based therapies. Previous studies have reported on short-term culturing of cardiac tissue slices. However, extensive electrophysiologic characterization after several days is lacking from these reports [15]–[17]. Therefore, in the present study, we investigated the (immuno) histologic and electrophysiologic properties of 6–8 days old cultured intact cardiac tissue (organ explant culture). We compared outcomes to both freshly isolated cardiac tissue and to monolayers to address the following questions. (1) Are organ explant cultures viable for 6–8 days? (2) How do the (immuno) histologic and electrophysiologic properties differ among these *in vitro* systems? (3) Can organ explant cultures be transduced by lentiviral vectors? (4) Can organ explant cultures functionally couple to spontaneously beating neonatal rat ventricular cardiomyocytes?

## Materials and Methods

### 2.1 Preparation of organ explant cultures and monolayers

All animal experiments were approved by the local Animal Experiments Committee (Academic Medical Center, University of Amsterdam; DCA102499) and carried out in accordance with national and institutional guidelines.

#### Organ explant cultures

To obtain organ explant cultures, 2-day old Wistar rats (Charles River) were decapitated, and hearts were excised and stored in cold HBSS (Gibco, #14025). Atria were removed and ventricles were separated into right ventricle, left ventricle, and septum. We either used these explants immediately for electrophysiologic studies or immunohistochemistry, or cultured them, endocardial side down, on drained collagen gels [18]. Organ cultures and gels were placed in an incubator at 37°C and 5% CO_2_. Organ cultures were left untreated overnight to allow for attachment to the gel, after which complete M199 medium (Gibco, #31150) was added, containing (mM) NaCl 117, KCl 5.3, CaCl_2_ 1.8, MgSO_4_ 0.8, NaHCO_3_ 26.2, and Na_2_HPO_4_ 1.0, supplemented with 1% fetal bovine serum, 1% penicillin/streptomycin 100× (Gibco, #15140–122), 5 μg/ml insulin, 5 μg/ml transferrin, 5 ng/ml selenium (ITS, Collaborative Research Inc., #40351), and 2 mM L-glutamine (Gibco, #25030–081). This medium, now called organ explant medium, was changed after 4 days and we conducted electrophysiologic and (immuno) histologic characterization after 6–8 days.

#### Monolayers of neonatal rat ventricular cardiomyocytes

To obtain monolayers of neonatal rat ventricular cardiomyocytes we used previously described protocols [19]. Briefly, after excision of the hearts and removal of the atria, ventricles were dissected into 4–6 fragments, which were left to rotate overnight at 4°C in HBSS (Gibco, #14170) without Ca^2+^ and Mg^2+^ but containing trypsin (1 mg/mL, USB, #22720). The next day, after inactivation of trypsin with culture medium, cells were dissociated in three steps in HBSS containing collagenase (1 mg/mL, Worthington #4176, 315 units/mg) at 37°C. Dissociation solutions were centrifuged and cells were resuspended in 10% culture medium. To separate fibroblasts from cardiomyocytes, cells were preplated for 2 hours in tissue-culture treated polystyrene T175 cell culture flasks at 37°C in 5% CO_2_. After two hours, non-adherent cells were collected and plated (0.6×10^6^ cells on 22 mm ø fibronectin (BD Biosciences, # 356009) coated glass coverslips). Neonatal rat ventricular cardiomyocytes were cultured at 37°C in 5% CO_2_ in monolayer medium, consisting of M199 (Gibco, #31150) containing (mM) NaCl 117, KCl 5.3, CaCl_2_ 1.8, MgSO_4_ 0.8, NaHCO_3_ 26.2, and Na_2_HPO_4_ 1.0, supplemented with 1% HEPES (Gibco, #15630–080), 5000 U/L penicillin-G (Sigma, #P7794), 2 mg/L vitamin B12 (Sigma, #V2876), 3,5 g/L glucose, 1% non essential amino acids (Gibco, #11140–050), 1% L-glutamine (Gibco, #25030–081), and either 10% (until day 3) or 2% (from day 3) fetal bovine serum (Gibco, #16140071). Also, several monolayers were cultured in organ explant medium to exclude an effect of culture medium composition on electrophysiologic characteristics. We conducted electrophysiologic and (immuno) histologic characterization after 6–8 days.

### 2.2 Histology

After embedding freshly isolated tissue and organ explant cultures in Tissue-Tek O.C.T (Sakura, # 4583), 10 μm thick sections were cut by microtome and stored at −20°C. For fixation of monolayers, glass coverslips with cells attached were washed with PBS twice and then covered in methanol (−20°C) for 2 minutes. Subsequently, the coverslips were washed with PBS 3 times. Fixed monolayers were kept at 4°C.

#### HE staining

For hematoxylin-eosin (HE) staining, tissue sections were thawed for 2 hrs and fixed in 4% PFA for 10 minutes. Afterwards, both the fixed sections and the previously fixed monolayers were washed with distilled H_2_O, stained with hematoxylin (Sigma) for 5 minutes, washed with H_2_O, and stained with eosin (Sigma) for 30 seconds. After washing with H_2_O, the preparations were shortly rinsed with 70% ethanol, 96% ethanol and 100% ethanol, after which they were left in xylol (Bufa) for 5 minutes and mounted with pertex (Histolab).

#### Immunohistologic staining

For immunohistochemistry, tissue sections, after thawing for 2 hrs, were rehydrated in PBS (Lonza, #BE17–516F) for 5 minutes. Monolayers were already stored in PBS. Afterwards, 0.2% Triton X-100 (Sigma, #T8787) in PBS was added for 1 hr to increase permeability of the cell membrane. After 4 washes with PBS, 2% BSA (MP Biomed) in PBS was added for 30 minutes. The primary antibody, dissolved in PBS containing 10% normal goat serum (Gibco, #16210–064), was left to incubate overnight. The next day, sections were washed with PBS. Tissue sections were incubated with secondary antibodies and nucleic acid staining (diluted in PBS containing 10% normal goat serum) for 2 hours. Sections were sealed with glycerol: PBS (1∶1) and stored at 4°C. Primary antibodies used are anti-connexin-43 mouse monoclonal (1∶100; BD Transduction; #610061), anti-α-smooth muscle actin mouse monoclonal (1∶1000; Sigma, #A2547), anti-α-actinin mouse monoclonal (1∶1000; Sigma; #A7811). Secondary antibodies used were Alexa-fluor antibodies diluted 1∶250, nuclear staining used was sytox orange (Invitrogen; #S11368, 1∶1000). The specificity of secondary antibodies was verified by incubating control sections with secondary antibodies only. Sections treated this way did not show any non-specific staining.

### 2.3 Intracellular recordings

To assess RMP, intracellular microelectrode recordings were made of tissue and cell cultures at 37°C in Tyrode's solution, which contained (mM) NaCl 140, KCl 5.4 mM, CaCl_2_ 1.8, MgCl_2_ 1.0, glucose 5.5, HEPES 5.0 (pH 7.4 with NaOH). Microelectrodes were filled with 3 M KCl, and tip resistance was 15–35 MΩ. A reference electrode was placed in the tissue bath. Action potentials were recorded at 16 kHz using a data acquisition system (Biosemi Actiview), and analyzed using custom-made software based on Matlab (Mathworks). We characterized tissue by (1) the presence of spontaneous beating activity, (2) the presence of phase 4 depolarization, defined as a diastolic depolarization rate of at least 5 mV/s measured over the 50 ms interval starting at the maximal diastolic potential (MDP), and (3) MDP or RMP (MDP in case of preparations showing phase 4 depolarization; RMP in case of quiescent tissue). Action potential amplitude, action potential duration (APD), and upstroke velocity were not measured due to difficulty keeping the microelectrode intracellular during contraction, especially in organ cultures.

### 2.4 Optical mapping

To assess conduction velocity (CV) and action potential properties, optical mapping of tissue and cell cultures was done. After exposure to 15 μM di-4-ANEPPS (Invitrogen, #D1199) in EBSS (Gibco, #24010) for 7.5 minutes, explants were placed in an optical mapping set-up in Tyrode's solution (37.0±0.5°C) containing (mM) NaCl 140, KCl 5.4 mM, CaCl_2_ 1.8, MgCl_2_ 1.0, glucose 5.5, HEPES 5.0 (pH 7.4 with NaOH) and blebbistatin 10 μmol/L (Tocris Bioscience, #1760). Six power light emitting diodes (5 W per diode, band-pass filtered 510±20 nm) delivered excitation light. Emission fluorescence (filtered >610 nm) was transmitted through a tandem lens system on a Complementary Metal Oxide Semiconductor chip (Micam Ultima) with a spatial resolution of 100×100 pixels. Data acquisition was carried out with a sample frequency of 1 kHz for freshly isolated tissue and organ explant cultures, and 0.5 kHz for monolayers (because of lower fluorescence intensity in monolayers). Data were analyzed using custom made software based on Matlab (Mathworks). We characterized tissue by (1) the presence of spontaneous beating activity, (2) CV, (3) anisotropy ratio, (4) maximal upstroke velocity, (5) APD at 20%, 50%, 70%, and 90% repolarization (APD20, APD50, APD70, APD90), and (6) the ability to stimulate tissue at different cycle lengths (CLs) varying from 500 ms to 100 ms. CV and maximal upstroke velocity were measured at a pacing CL of 500 ms. CV was calculated from activation times – based on time of maximal upstroke velocity – recorded at 2 sites along a line perpendicular to the isochronal lines, when stimulated at 2 times stimulation threshold at a cycle length of 500 ms. The anisotropy ratio was defined as maximal CV divided by CV measured perpendicular to maximal CV. Maximal upstroke velocity was determined in normalized action potentials – baseline was set to 0 and maximal amplitude to 100 – and defined as the maximal rise in percentage of action potential amplitude per ms (d(%APA)/dt*_max_*). Because of the lower sample frequency, this could not be measured in monolayers.

### 2.5 Gene and cell therapy

A lentiviral *Egfp* expression vector (pRRL-cPPT-CMV-X2-IRES-Egfp-PRE-SIN lentiviral transfer vector, from here on called LV-*Egfp*) was made as described previously [20]. Virus was produced via cotransfection of HEK293T cells, and titrated by detection of EGFP expression 3 days after transduction of HeLa cells.

To test the feasibility of gene transfer we injected virus LV-*Egfp* (1×10^6^ IU) into organ explant cultures at day 0, before attachment of the tissue to the gel, in the presence of dextran (10 Μg/mL; Sigma, #D9885). After overnight incubation, we added complete M199 medium.

To test the utility of cell therapy, freshly isolated neonatal rat ventricular cardiomyocytes were seeded on top of one-day old organ explant cultures (0.3×10^6^ cells per explant) to form a spontaneously beating confluent monolayer. Spontaneous beating of organ explant cultures was measured via microelectrode measurements and optical mapping.

### 2.6 Statistical analysis

Data are presented as mean ± SEM. Statistical tests used include Student's *t*-test and one-way ANOVA for normally distributed continuous data and Pearson's chi-squared test or Fisher's exact test for categorical data. *P*<0.05 was considered statistically significant. SPSS 18 was used for statistical analysis.

## Results

### 3.1 Histology

**Figure 1:**
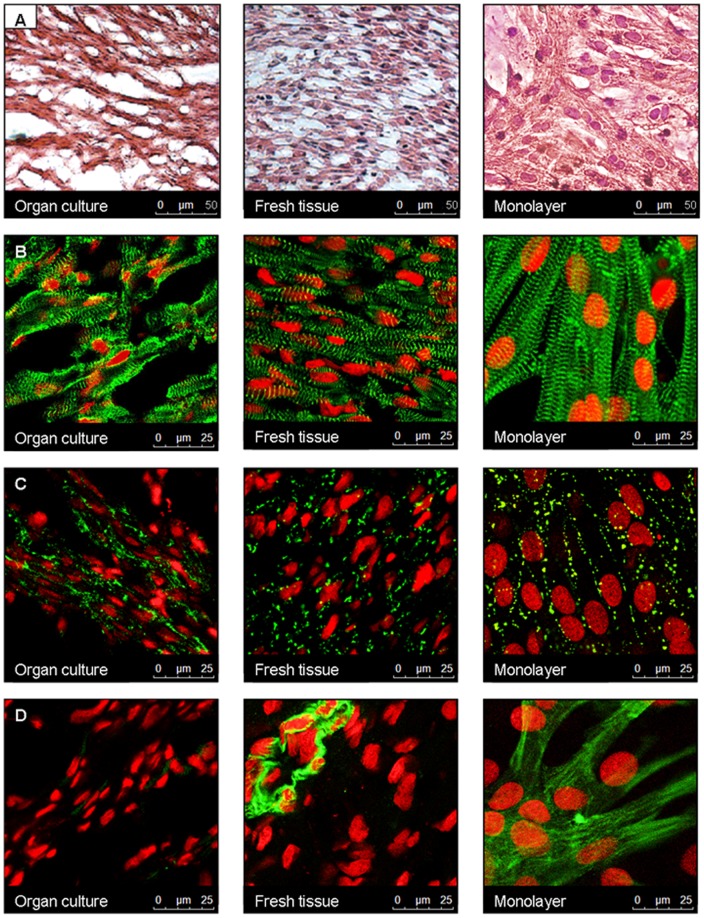
Organ explant cultures are (immuno) histologically similar to freshly isolated tissue. From left to right: organ explant cultures, freshly isolated tissue, and monolayers. **A,** Hematoxylin-eosin staining. **B,** Immunohistochemistry for α-actinin (green). **C,** Immunohistochemistry for connexin-43 (green). **D,** Immunohistochemistry for α-smooth muscle actin (green). Nuclei are stained with sytox orange (red).

Hematoxylin-eosin (HE) stained organ explant cultures had a more loose tissue structure than freshly isolated tissue, but maintained a longitudinal fiber orientation. In contrast, monolayers did not show a longitudinal fiber orientation (Figure 1A). Immunohistochemistry for the cytoskeletal protein α-actinin, which is localized to the Z-discs, showed a striated pattern in all groups (Figure 1B). Also, immunostaining for the gap-junction protein connexin-43 showed a similar pattern in all 3 groups, i.e., localization not only to the intercalated discs, but along all cell borders (Figure 1C). Expression of α-smooth muscle actin, a marker for myofibroblasts and smooth muscle cells, was abundantly present throughout the whole preparation in monolayers, while this was not the case in both freshly isolated tissue and organ explants cultures. In the example of freshly isolated tissue shown in Figure 1D, α-smooth muscle actin was present in vascular smooth muscle cells.

### 3.2 Intracellular recordings

**Figure 2:**
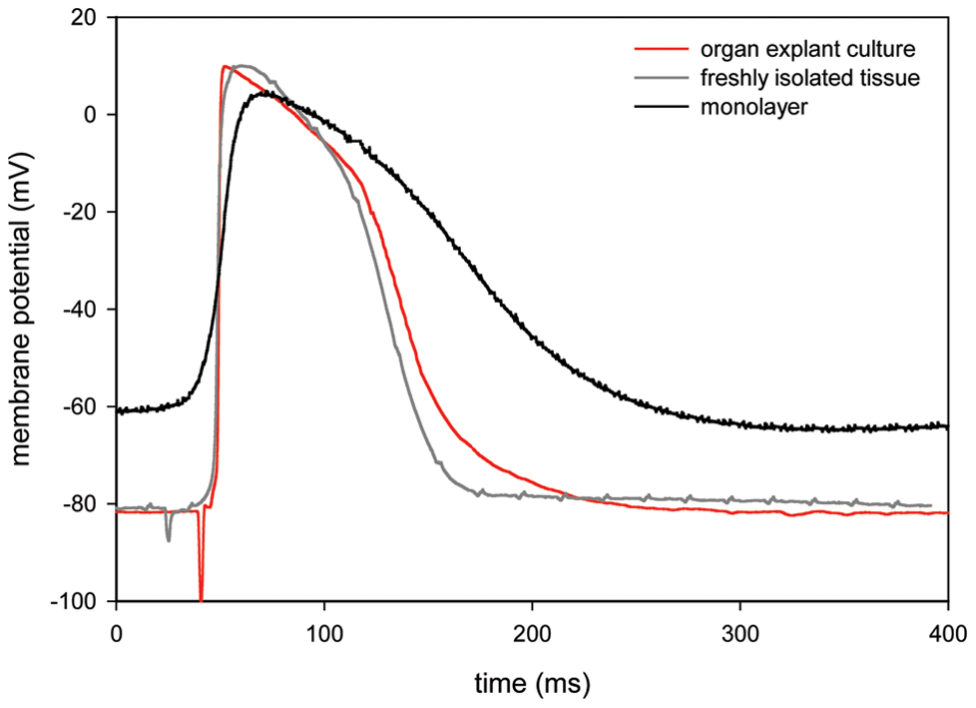
Typical examples from intracellular recordings. Typical examples of action potentials as measured with intracellular recordings when stimulated at 500 ms cycle length.

Table 1 shows results from microelectrode measurements; Figure 2 depicts typical examples of stimulated action potentials when stimulated at 500 ms CL. Twenty percent of organ explant cultures (n = 10; 4 left ventricle, 3 right ventricle, 3 septum) showed spontaneous beating activity. This proportion was similar to freshly isolated tissue (17% [n = 6; 2 left ventricle, 2 right ventricle, 2 septum], p = NS). In contrast, 82% of monolayers (n = 17) beat spontaneously (p<0.05 vs. both other groups). Accordingly, we observed phase 4 depolarization in 20% of measurements in organ explant cultures, never in freshly isolated tissue, and in 67% of the monolayers (p<0.05 vs. both other groups).

**Table 1:** Summary data from intracellular recordings.

	Organ explant culture (n = 10)	Freshly isolated tissue (n = 6)	Monolayers (n = 17)
**Spontaneous beating activity**	2 (20%) *	1 (17%) ^†^	14 (82%) *^†^
**Phase 4 depolarization**	2 (20%) *	0 (0%) ^†^	11 (65%) *^†^
**RMP/MDP (mV)**	−83.9±4.4 *	−80.5±3.5 ^†^	−60.9±4.3 *^†^

Values are n (%) or mean ± standard error of the mean. RMP, resting membrane potential; MDP, maximal diastolic potential. *−* or †−† statistically different from each other, p<0.05.

RMP of organ explant cultures was similar to freshly isolated tissue (−83.9±4.4 mV and −80.5±3.5 mV, respectively, p = NS), while being more depolarized in monolayers (−60.9±4.3 mV; p<0.05).

### 3.3 Optical mapping

Table 2 summarizes results from optical mapping. Similar to intracellular recordings, the proportion of spontaneous beating activity of organ explant cultures was comparably low as in freshly isolated tissue (36% [n = 22; 7 left ventricle, 7 right ventricle, 8 septum] and 33% [n = 12; 4 left ventricle, 4 right ventricle, 4 septum], respectively, p = NS), but much higher in monolayers (89% [n = 26], p<0.05 vs. both other groups). In spontaneously beating preparations, cycle length did not differ between organ explant cultures, freshly isolated tissue and monolayers (468±97 ms, 677±85 and 582±60 ms, respectively). Spontaneous activity in organ explant cultures and freshly isolated tissue never originated from the dissection border of the tissue.

**Table 2:** Summary data from optical mapping.

	Organ explant culture (n = 22)	Freshly isolated tissue (n = 12)	Monolayers (n = 26)
**Spontaneous beating activity**	8 (36%) *	4 (33%) ^†^	23 (89%) *^†^
**CL (ms)**	468±97	677±85	582±60
**CV (cm/s)**	18.2±1.0 *	18.6±1.0 ^†^	24.3±0.7 *^†^
**Anisotropy ratio**	2.5±0.1 *	2.1±0.2 ^†^	1.2±0.04 *^†^
**d(%APA)/dt** *_max_*	46.8±2.4	50.4±1.9	

Values are n (%) or mean ± standard error of the mean. CL, cycle length; CV, conduction velocity; d(%APA)/dt*_max_*_,_, maximal upstroke velocity. *−* or †−† statistically different from each other, p<0.05.

**Figure 3:**
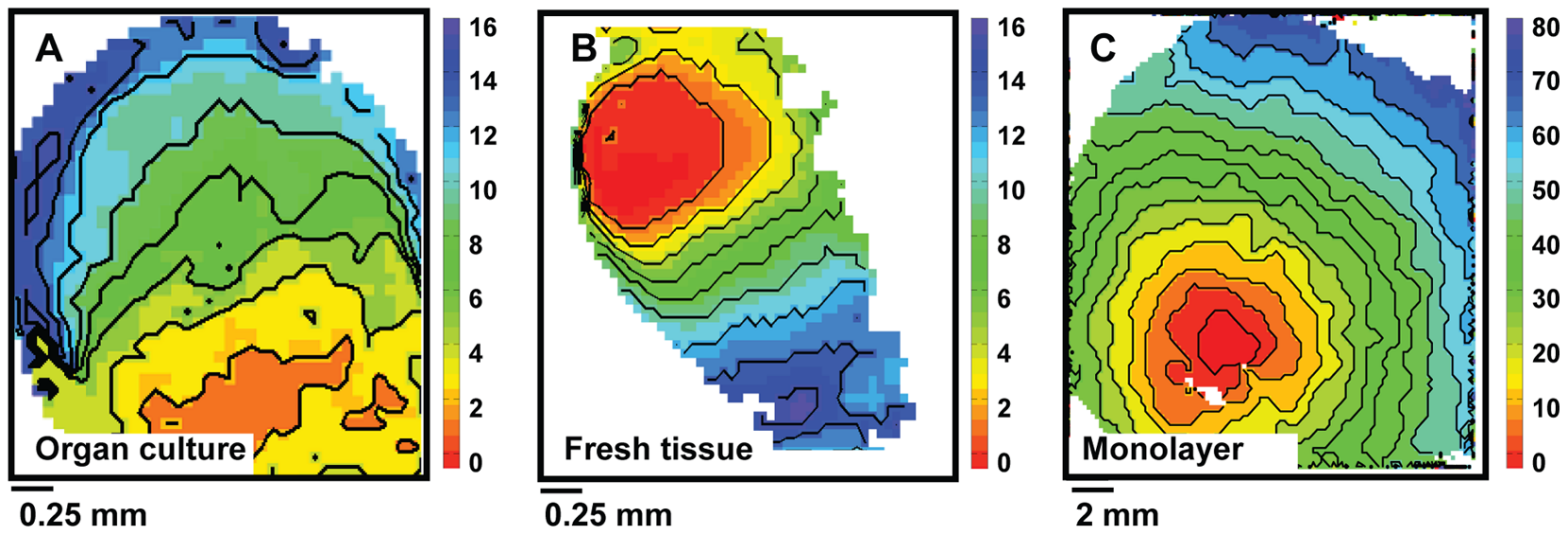
Isochronal activation maps. Isochronal activation maps constructed from the moment of maximal action potential upstroke velocity. Numbers are activation times in ms. **A,** Organ explant culture. **B,** Freshly isolated tissue. **C,** Monolayer.

Maximal CV of organ explant cultures was similar to that of freshly isolated tissue (18.2±1.0 cm/s and 18.6±1.0 cm/s, respectively, p = NS), but lower than in monolayers (24.3±0.7 cm/s, p<0.05 vs. both other groups). Electrical conduction remained anisotropic in organ explant cultures (anisotropy ratio 2.5±0.1), similar to freshly isolated tissue (2.1±0.2, p = NS), while conduction in monolayers was isotropic (1.2±0.04, p<0.05 vs. both other groups). Figure 3 shows typical examples of isochronal activation maps, based on time of d(%APA)/dt*_max_* of different types of tissue studied.

We did not observe a difference in d(%APA)/dt*_max_* between organ explant cultures and freshly isolated tissue (46.8±2.4 and 50.4±1.9 %APA/ms, p = NS).

**Figure 4:**
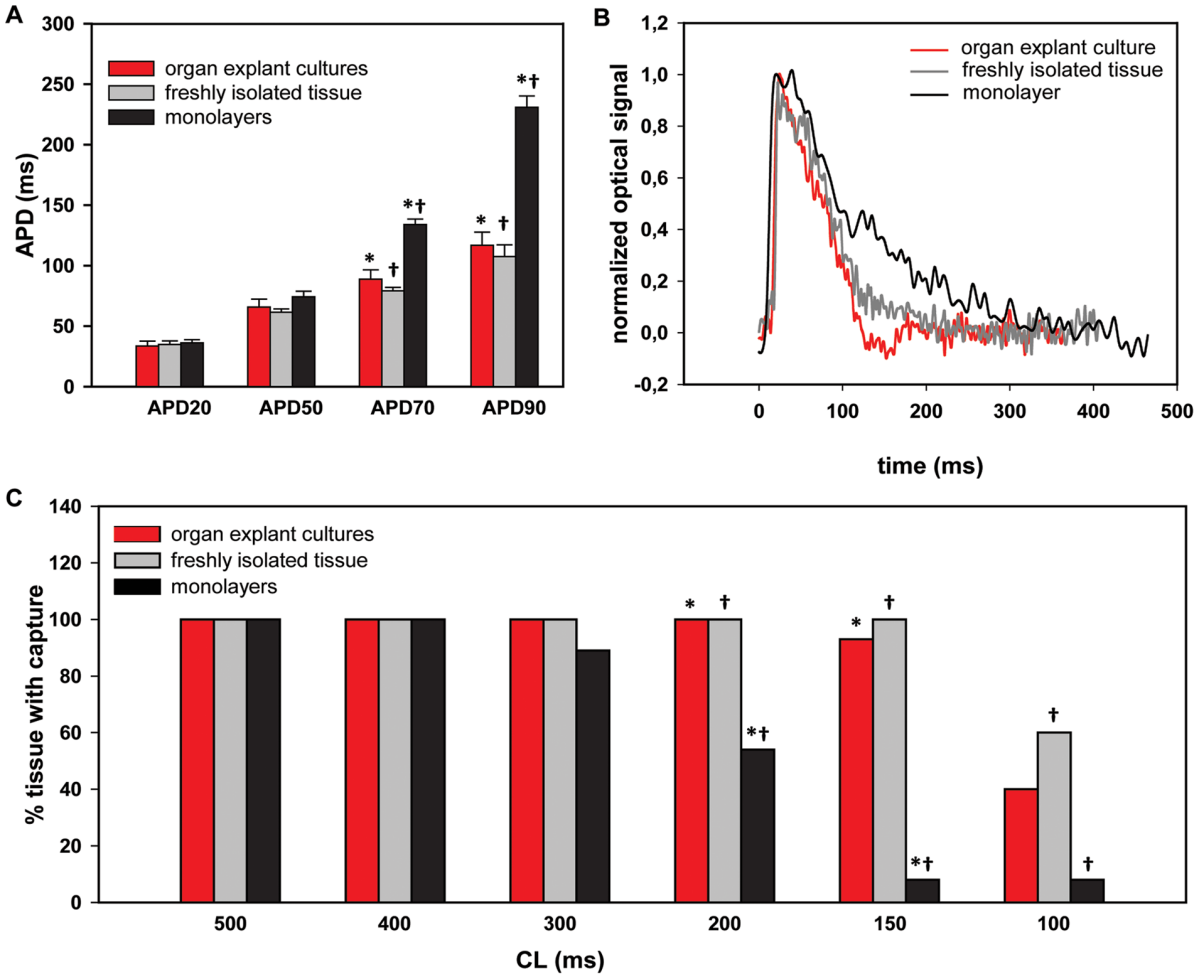
Action potential duration and capture at different cycle lengths. **A,** Action potential duration at 20%, 50%, 70% and 90% repolarization (ms; mean ± SEM). **B,** Typical example of action potentials as seen with optical mapping. **C,** Percentage of tissue showing propagated action potentials when stimulated at 500, 400, 300, 200, 150, and 100 ms cycle length. *−* or †−† statistically different from each other, p<0.05.

While APD20, APD50, APD70, and APD90 did not differ between organ explant cultures and freshly isolated tissue (33.6±4.0, 35.0±2.9 for APD20; 65.8±6.5, 61.5±2.8 for APD50; 88.8±7.8, 79.1±2.9 for APD70; and 116.8±10.9 and 107.5±9.8 ms for APD90, respectively), APD70 and APD90 were increased in monolayers (134.0±4.5 and 230.8±9.5 ms; p<0.05 vs. both other groups; Figure 4A).

When stimulated at 500, 400 and 300 ms CL, all groups showed similar capture rates (n = 15 organ explant cultures, n = 10 freshly isolated tissue, n = 26 monolayers; Figure 4C). When stimulated at 200 ms CL, only 54% of monolayers were captured, as compared to 100% of organ explant cultures and 100% of freshly isolated tissue (p<0.05 vs. both other groups). This difference was more prominent at shorter CLs, e.g., at 150 ms (93% of organ explant cultures, 100% of freshly isolated tissue, 8% of monolayers) and at 100 ms (40% of organ explant cultures, 60% of freshly isolated tissue, 8% of monolayers).

**Figure 5:**
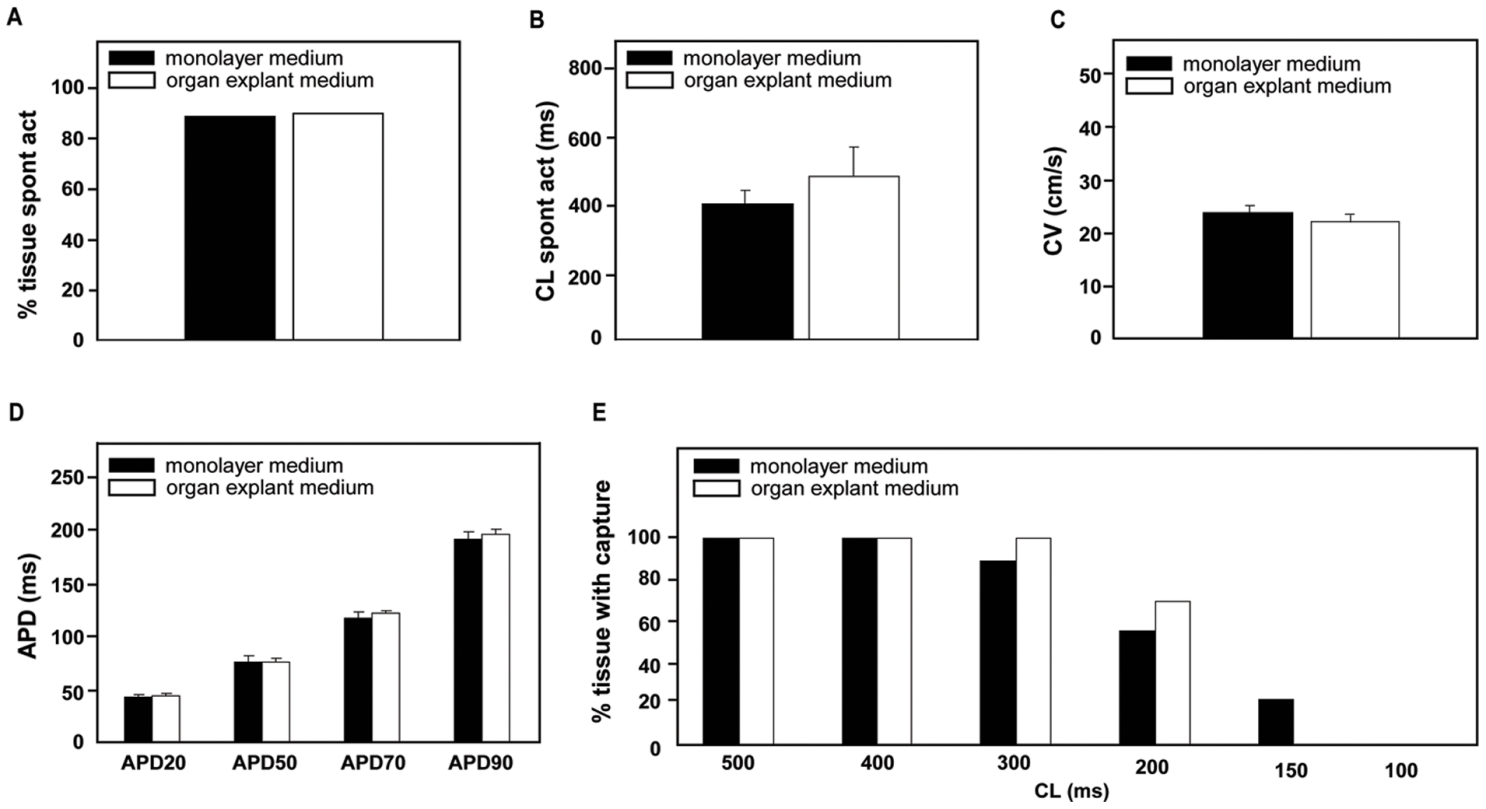
Optical mapping results comparing monolayers cultured in monolayer medium or organ explant medium. **A,** Percentage of monolayers showing spontaneous beating activity. **B,** Cycle length of spontaneously beating monolayers (ms; mean ± SEM). **C,** Conduction velocity (cm/s; mean ± SEM). **D,** Action potential duration at 20%, 50%, 70% and 90% repolarization (ms; mean ± SEM). **E,** Percentage of monolayers showing propagated action potentials when stimulated at 500, 400, 300, 200, 150, and 100 ms cycle length.

We further established that the composition of the culture medium was not of influence on monolayer characteristics (Figure 5). Monolayers grown in organ explant medium (n = 10) showed electrophysiologic characteristics similar to monolayers grown in monolayer medium (n = 9). Both groups showed a high occurrence of spontaneous beating activity (89% vs 90%), similar CL of spontaneous beating activity (399±37 vs 479±86), and no differences in CV, APD, and capture when stimulated at different CLs (all p = NS).

### 3.4 Gene and cell therapy

**Figure 6:**
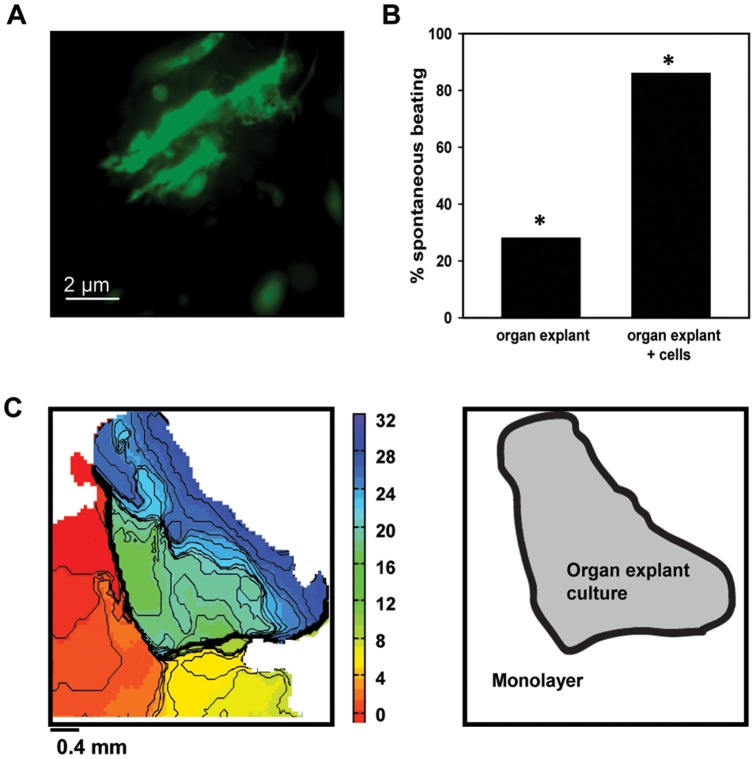
Organ explant cultures can be used to study the effects of gene and cell therapy. **A,** EGFP expression in organ explant cultures transduced with LV-*Egfp*. **B,** Percentage of preparations showing spontaneous beating, comparing organ explant cultures alone with organ explants co-cultured with spontaneously beating neonatal rat ventricular cardiomyocytes. **C,** Isochronal activation map of organ explant culture with spontaneously beating neonatal rat ventricular cardiomyocytes, constructed from the moment of maximal action potential upstroke velocity. Spontaneous beating originates from the monolayer, with capture of organ explant culture. Numbers are activation times in ms.*−* statistically different from each other, p<0.05.

After establishing that electrophysiologic and histologic properties of organ explant cultures were highly comparable with freshly isolated tissue, we next tested how well organ explant cultures were suitable for lentiviral gene transfer. Injection of LV-*Egfp* into the center of organ explant cultures (n = 4) resulted in expression of EGFP at the site of injection (Figure 6A).

Finally, we investigated whether organ explant cultures may be useful in evaluating the effects of cell therapy, by testing their capability to couple to other cardiomyocytes. Here we found the addition of 0.3×10^6^ spontaneously active neonatal rat ventricular cardiomyocytes to increase the percentage of spontaneously beating organ explant cultures to 86% (CL 605.8±70.8 ms; Figure 6B). In comparison, above reported unmodified organ explant cultures exhibited spontaneous beating only in 28% (n = 32) of the samples (p<0.05). Figure 6C shows a typical example of an isochronal activation map, with spontaneous beating originating from the monolayer of isolated neonatal rat ventricular cardiomyocytes, with capture of the organ explant culture.

## Discussion

Interpretation of the results from *in vitro* studies on gene therapies in culture models of neonatal rat ventricular cardiomyocytes is often confounded by the occurrence of a partial loss of the native physiologic properties. In the present study, we investigated whether a new *in vitro* model of long-term organ explant culture retained its *in vivo* properties, and whether this model is suitable for studying gene- and cell-based therapies. Using (immuno) histochemical and electrophysiologic analysis, we demonstrated that long-term heart organ explant cultures were viable up to 8 days in culture, while still retaining multiple critical properties of freshly isolated cardiac tissue. Cardiomyocytes in organ explant culture retained their longitudinal fiber orientation, while expression patterns of the structural proteins α-actinin, connexin-43, and α-smooth muscle actin were similar to freshly isolated cardiac tissue. Also, the electrophysiologic properties were largely preserved in organ explant cultures. As in freshly isolated cardiac tissue, most of the cultures did not show spontaneous beating activity and had stable RMP of approximately −85 mV. In addition, CV, anisotropy ratio, maximal upstroke velocity, and APD were unaltered.

In comparison, alterations in (electro) physiologic properties in monolayers were more pronounced. Immunoreactivity for α-smooth muscle actin was abundantly present throughout monolayers, indicative for the presence of myofibroblasts. Moreover, monolayers often beat spontaneously, and showed a depolarized RMP, an increased presence of slow diastolic depolarization, a loss of anisotropic conduction, and a prolonged APD as compared to freshly isolated tissue. Also, while organ explant cultures could be paced at frequencies similar to freshly isolated tissue, monolayers were more frequently not captured at short CLs.

Finally, we demonstrated that organ explant cultures can be transduced with LV-*Egfp* (inferred from the expression of EGFP), and are capable of functional coupling to spontaneously beating neonatal rat ventricular cardiomyocytes.

### Origin of α-smooth muscle actin expression in monolayers

The expression of α-smooth muscle actin in organ explant cultures and freshly isolated tissue is related to the presence of smooth muscles in the vasculature [21]. In monolayers, the origin of the α-smooth muscle actin is less clear. While recent reports suggest that this is due to the presence of myofibroblasts [11], [12], it could also be the result of dedifferentiation of cardiomyocytes, since α-smooth muscle actin is also temporarily expressed in fetal rat cardiomyocytes [22]. Zhang et al. have shown that cultured cardiomyocytes can dedifferentiate and express early transcription factors, but they did not investigate the expression of α-smooth muscle actin in these cells [23]. *In vivo*, the expression of α-smooth muscle actin in both adult cardiomyocytes and myofibroblasts has been described in cardiac diseases such as atrial fibrillation and right ventricular pressure overload [24], [25].

### Myofibroblasts, MDP, and spontaneous activity

Several studies investigated the effects of different amounts of (myo) fibroblasts on MDP and spontaneous beating activity of monolayers [10], [26]. In these studies a preplating step was used to separate fibroblasts from cardiomyocytes, and bromodeoxyuridine was added to influence the growth of (myo) fibroblasts present in culture. Miragoli et al. described a decrease in MDP from approximately −80 mV to −50 mV when the amount of myofibroblasts increased. In that study, experiments were performed in 3−4 days old preparations. Since we want to use our model to further study the effect of lentiviral gene transfer after 6 days, we tried to decrease the number of myofibroblasts this way. However, in our experience, decreasing the amount of (myo) fibroblasts via preplating and bromodeoxyuridine was only effective for a limited amount of time. Alternatively, pharmacologic interventions such as cytochalasin D or latrunculin B have been shown to disturb actin function, restore membrane potential and stop spontaneous beating in cell pairs of myofibroblasts and neonatal rat ventricular cardiomyocytes [12]. Yet, we did not attempt to use this type of interventions as they do not prevent abundant presence of myofibroblasts and membrane potentials remain relatively depolarized at approximately −74 mV.

Another factor that can contribute to spontaneous beating in cultured cells is a decreased inward rectifier current (I_K1_), which has been shown in both adult rat and rabbit ventricular myocytes [8], [27]. This progressive decrease in I_K1_ was correlated to a depolarization of the RMP, and could thus also influence the occurrence of spontaneous beating.

Even in the absence of myofibroblast and with a RMP of approximately −84 mV, some of the organ explant cultures and freshly isolated tissues showed spontaneous beating activity. Spontaneous beating was observed in some preparations where we did not detect phase 4 depolarization. This likely occurred in settings where the impalement site was not at the origin of spontaneous membrane depolarization. We showed that the beating activity in organ explant cultures and freshly isolated tissue did not originate from the dissection border of the tissue, excluding tissue damage as a causative factor in our preparations. Since part of the conduction system is still present in freshly isolated tissue and organ explant cultures, it is possible that spontaneous beating originated from these cells.

### Conduction velocity

CV of freshly isolated tissue was relatively low (18.2±1.0 cm/s) compared to reported values for adult rat ventricle (66±8 cm/s) [28]. Only few papers have reported CV in neonatal rat heart [29]–[31], and showed values ranging from 21 cm/s in 2-day old rats to 31 cm/s in 10-day old rats. This increase in CV with increasing age was found in several species [32], [33].

We found that CV in monolayers was higher than in organ explant cultures and freshly isolated tissue. This was somewhat surprising, given the fact that organ explant cultures had electrophysiologic properties and tissue architecture that were more favorable (i.e., conducive to high CV) than monolayers. CV has multiple determinants, whose net contributions are difficult to predict. For instance, fiber direction, a determinant of CV [34], appears more favorable for organ explant cultures than monolayers, as longitudinal cell orientation (associated with large CV in the longitudinal direction) was preserved in organ explant cultures, but not in monolayers. Paradoxically, tissue geometry may also provide a potential cause for the lower CV in organ explant cultures. Since a monolayer is a 2-dimensional network, the activation wavefront must travel in a horizontal plane, i.e., the plane that is measured by optical mapping. However, in organ explant cultures, the loose 3-dimensional tissue structure may give rise to activation wavefronts that travel outside the planes of optical measurement. In that case, the CV will be underestimated.

Another factor that can influence CV is the magnitude of the upstroke velocity [35]. However, since the sample time was 2 ms in optical mapping of monolayers, we could not measure upstroke velocity and compare this to organ explant cultures and freshly isolated tissue, as these were mapped with a sample time of 1 ms.

A possible contributor to higher CV in monolayers may be larger cell size. While we did not quantify cell surface area, in both HE staining and immunohistochemistry, cell surface area in monolayers appeared to be larger, which may partly explain larger CV [36], [37]. A final determinant of CV to consider is cell-to-cell coupling [36]. However, this determinant is not likely to explain differences in CV, as connexin-43 expression was similar in all groups; being distributed along all cell borders, instead of being localized to the intercalated discs as in adult tissue [38]. We did not investigate Cx45 expression and therefore cannot rule out potential differences among the individual groups. Although the presence and role of connexin-45 during embryonic development have been well established [39], its role in neonatal and adult tissue seems limited, as it has consistently been found in very low quantities in both neonatal and adult heart [40]–[42]. Furthermore, in cell pairs of neonatal rat ventricular cardiomyocytes there is a linear correlation between gap junction conductance and connexin-43 immunosignal [43]. We therefore like to suggest that potential differences in connexin-45 are unlikely to explain the differences in CV in our study. Nevertheless, if there is a difference in expression of connexin-45 in monolayers as compared to freshly isolated tissue and organ explants cultures, this could influence CV, as mixed channels containing both connexin-43 and -45 have a lower channel conductance [44]–[46]. This lower gap junction conductance is expected to decrease CV [47].

While CV is higher in monolayers than organ explant cultures, freshly isolated tissue has a similar CV to organ explant cultures, which leads us to believe that the organ explant cultures in this study showed a CV within the normal range for neonatal rat ventricular tissue.

### Action potential duration

The APDs of freshly isolated neonatal rat ventricle and monolayers reported vary between different studies, and the values as reported in this paper are consistent with previously published studies [48]–[50]. The prolonged APD seen in monolayers as compared to freshly isolated tissue can be explained by their depolarized MDP. Due to this depolarization, both the transient outward current (I_to_) and the rapid component of the delayed rectifier current (I_Kr_) are inactivated more strongly [51]–[53]. The resulting decrease in repolarizing currents is expected to prolong APD. Bursac and colleagues indeed showed that blocking I_to_ with 4-aminopyridine in freshly isolated tissue strongly increased the APD, while blocking I_to_ had no effect on the APD in monolayers [49].

### Stimulation at different cycle lengths

Often properties of monolayers are studied at a 2 Hz pacing rate. Since neonatal rat hearts beat at around 350 beats per minute (CL∼170 ms; [54], [55]), we also investigated capture at shorter CLs. While both organ explant cultures and freshly isolated tissue could still be paced with a CL of 150 ms, capture was frequently lost in monolayers at CLs of around 200 ms. This finding is in accordance with the longer APD found in monolayers.

### Conclusion

In conclusion, we developed a method of organ explant culture that provides an *in vitro* model of cardiac tissue that is structurally and electrophysiologically similar to freshly isolated tissue. In accordance with its tissue architecture, (near) absence of spontaneous beating and myofibroblasts render this model superior to the well established model of neonatal rat ventricular cardiomyocytes cultured in monolayer. Because the cultured organ explant model also appeared readily modified by methods of gene and cell therapy, this model may offer a powerful screening tool for the evaluation of biological approaches aimed at treatment of cardiovascular disease.
